# Case Report: Omalizumab-associated hair loss: a case of eyebrow alopecia areata, literature review and FAERS database analysis

**DOI:** 10.3389/fmed.2025.1605826

**Published:** 2025-06-27

**Authors:** Qian Wang, Hua Lei, Ge Yang, Ying Li, Wei Liu, Lixia Zhang, Xiyuan Zhou

**Affiliations:** Institute of Dermatology and Venereology, Sichuan Provincial People’s Hospital, University of Electronic Science and Technology, Chengdu, China

**Keywords:** omalizumab, hair loss, alopecia, side effect, FAERS database

## Abstract

Hair loss is a rare adverse reaction associated with omalizumab, with limited literature reports. The incidence, underlying mechanisms, and clinical characteristics of omalizumab-associated hair loss have not been clarified. We report a 52-year-old female with chronic spontaneous urticaria (CSU) who developed significant loss of eyebrows after 12 weeks of omalizumab treatment at 300 mg per 4 weeks. The diagnosis of alopecia areata (AA) was confirmed through dermoscopic examination and clinical manifestation. While maintaining omalizumab treatment for CSU, topical 0.03% tacrolimus ointment was initiated for treatment of AA. Regrowth of eyebrows was observed at 28 weeks of omalizumab treatment. We then performed a literature review, analyzing eight patients from five articles and one conference abstract, and concurrently analyzed data from 756 cases reported in the FDA adverse event reporting system (FAERS) database of patients developing alopecia after omalizumab use. Our findings suggest that omalizumab may induce alopecia, particularly among females and individuals aged 18–60 years. However, establishing a direct cause-effect relationship between alopecia and the drug remains challenging. AA may be the more uncommon type of omalizumab-associated hair loss, though it may be a transient phenomenon. Even if the incidence of this adverse effect is low, it warrants the clinician’s attention. Improved recognition of omalizumab-associated hair loss can optimize pretreatment preparation and patient counseling.

## 1 Introduction

Omalizumab is a recombinant humanized IgG1 monoclonal antibody that blocks the interaction between free IgE and FcεRI receptors on the surface of mast cells and basophils by binding to free IgE, thereby reducing the reactivity of these effector cells and blocking the inflammatory cascade. Omalizumab also blocks IgE-dependent antigen presentation and participates in the inhibition of the Th2-mediated inflammatory cascade, potentially preventing IgE-mediated allergic reaction in multiple ways (1). Omalizumab is currently approved for the treatment of moderate to severe persistent allergic asthma and chronic spontaneous urticaria (CSU) resistant to H1-antihistamine therapy. Furthermore, omalizumab has also been attempted in diseases such as allergic rhinitis (2), bullous pemphigoid (3), and atopic dermatitis (4), demonstrating its potential therapeutic value in these conditions.

Omalizumab has a favorable safety and tolerability profile, with several clinical studies confirming that the incidence of adverse reactions associated with the drug is comparable to placebo (5–7). A recently published meta-analysis also showed that omalizumab use was not associated with the increased risk of any serious adverse events (SAEs), including but not limited to various infectious diseases, further confirming its general safety (8). Nasopharyngitis, sinusitis, viral upper respiratory tract infection, headache, arthralgia and cough are common adverse reactions (9, 10). The package insert mentions some side effects without enough available data to estimate their frequency, such as idiopathic thrombocytopenia, myalgia, joint swelling, and hair loss. Alopecia is a special phenotype in drug safety evaluations. Anagen effluvium, telogen effluvium, alopecia areata (AA), and scarring alopecia may all potentially related to medications. The underlying mechanisms of medication-induced hair loss are complex and may involve abnormalities in the normal hair cycle and dysregulation of immune pathways.

Here, we report a patient treated with omalizumab for CSU who developed AA only involving eyebrows after 12 weeks of first exposure. AA is a multifactorial autoimmune disease with clinical manifestations ranging from hair loss in well-defined patches to diffuse or total hair loss. Although the mechanism and pathophysiology of AA remain unclear, they may be related to genetics, immune, environmental factors, etc., and medications also potentially serving as a contributing trigger. Through a systematic literature review, we retrieved a total of eight patients who developed alopecia during treatment with omalizumab. It has been reported in a recent literature that immunomodulatory agents and monoclonal antibodies represented the highest proportion of adverse event (AE) reports for alopecia (11). Given the limited literature reports on omalizumab-associated hair loss, we further analyzed 756 cases of alopecia following omaluzumab use in FAERS database between 2004 and 2024 to assess the epidemiological profile of this safety signal. Our aim was to use this AA case as an entry point to explore the potential association between omalizumab and alopecia, possible underlying mechanisms, and to call attention to this phenomenon in clinical practice.

## 2 Case presentation

**Figure 1:**
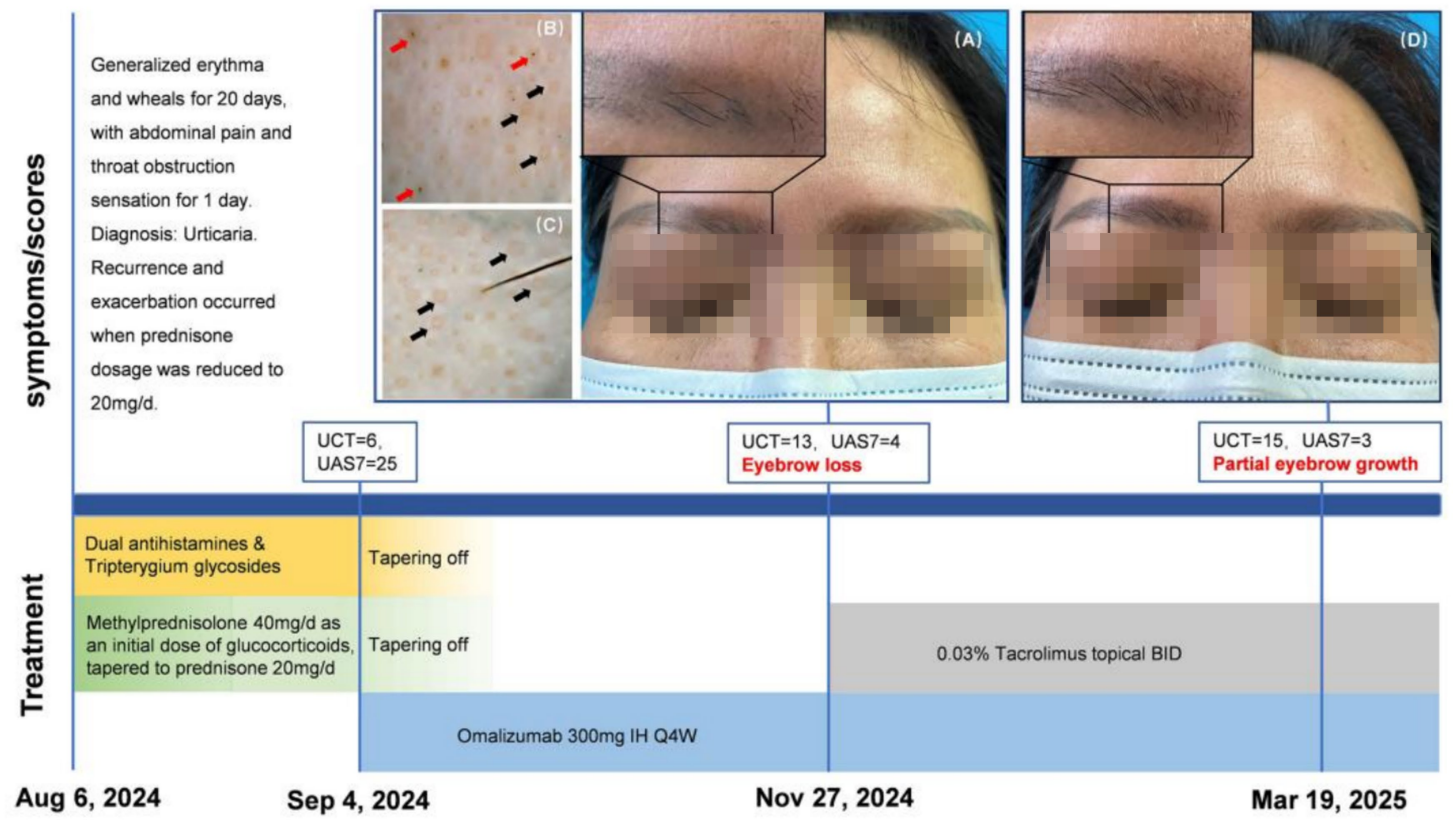
Clinical timeline and therapeutic outcomes of urticaria management, omalizumab-associated eyebrow alopecia areata development and treatment response. **(A–C)** Clinical manifestation and dermoscopic findings of eyebrow loss after 12 weeks of treatment with omalizumab. Clinical photograph demonstrating bilateral partial hair loss of eyebrows **(A)**. Dermoscopy revealed characteristic features of alopecia areata, including black dots (red arrows) and yellow dots (black arrows) **(B,C)**. **(D)** Follow-up at 16 weeks after initiation of topical 0.03% tacrolimus ointment alongside continued omalizumab treatment (at 28 weeks of omalizumab use). Regrowth of eyebrows was observed, with partial restoration of hair density.

A 52-year-old woman with generalized erythma and wheals for 20 days was admitted to our department in August 2024. She reported abdominal pain, tightness of breath, and throat obstruction sensation the day before admission. She was diagnosed for urticaria. She had a history of eyebrow tattooing more than 20 years ago and denied personal and family history of atopic diseases. Auxiliary examination showed: leukocytes to 10.7 × 10^9^/L (reference range: 3.5–9.5 × 10^9^/L), C-reactive protein (CRP) to 6.60 mg/L (reference range: ≤5 mg/L), D-dimer to 0.86 mg/L FEU (reference range: 0–0.55 mg/L FEU). No significant abnormalities were observed in the ANA spectrum, erythrocyte sedimentation rate (ESR), liver and renal function, procalcitonin (PCT), complement, immunoglobulin E (IgE), and urinalysis. Rupatadine 10 mg qd, fexofenadine 180 mg qn, tripterygium glycosides 20 mg tid, and methylprednisolone 40 mg qd were given after admission. The patient was discharged after 1 week treatment with improved condition. Then, the glucocorticoid dosage was gradually tapered. However, when the prednisone dose was reduced to 20 mg daily, urticaria recurred. At that time, the patient was still taking oral antihistamines and tripterygium glycosides. The diagnosis of CSU was established based on a disease duration exceeding 6 weeks and absence of identifiable triggers. After thorough communication with the patient, cyclosporine was declined due to concerns about adverse reactions. Consequently, omalizumab (300 mg/4 weeks) was initiated when the urticaria course approached 2 months. At baseline prior to omalizumab treatment, she had urticaria control test (UCT) score of 6 and urticaria activity score over 7 days (UAS7) of 25. Symptoms rapidly improved after the first dose of omalizumab. Over the following month, prednisone, tripterygium glycosides and antihistamines were gradually tapered and discontinued. After 12 weeks of omalizumab therapy, the patient achieved well-controlled disease with only occasional itching (UCT = 13, UAS7 = 4). However, she reported significant eyebrows loss. Physical examination revealed residual pigmentation, significant eyebrows thinning with alopecic patches, and scalp hair and eyelashes were not involved (Figure 1A); however, the result of the gentle pull test was negative. Dermoscopy showed obvious yellow dots and a few black dots on the eyebrow region, with no broken hairs or exclamation mark hairs (Figures 1B,C). She refused a skin biopsy. Based on clinical and dermoscopic findings, a diagnosis of AA was made. According to the Naranjo algorithm (12), the association between omalizumab and her new-onset AA was “possible.” Given that eyebrow tattooing minimized the cosmetic impact of eyebrow hair loss, she opted to continue regular omalizumab administration. Due to refusal of topical glucocorticoids, she was concurrently prescribed topical 0.03% tacrolimus ointment for AA. At 28 weeks of omalizumab treatment, she maintained stable CSU control (UCT = 15, UAS7 = 3) and hair regrowth was observed in the eyebrow region (Figure 1D).

## 3 Literature review

Five articles and one conference abstract reporting on eight cases of omalizumab related hair loss were identified and analyzed using the Google Scholar, SCOPUS, PubMed and Web of Science Core Collection databases (13–18), (see Table 1). Specific retrieval terms included: (omalizumab) or (anti-IgE) AND (alopecia) or (hair loss). The cohort comprised five women and three males, aged 14–70 years (35.75 ± 20.60). Age distribution included four patients under 30 years, two aged 30–50 years, and two over 50 years. Reasons for omalizumab use included urticaria in six cases (three of which were combined with angioedema), asthma and nasal polyps in one case, asthma with allergic rhinitis and allergic bronchopulmonary mycosis in one case. Comorbidities included atopic dermatitis with seasonal allergic rhinitis in one patient and thyroid disorders in another. Alopecia types comprised four cases of AA (including one case of alopecia universalis), three cases of telogen effluvium, and one case of hair loss without specifying the exact type. All of the AA cases occurred ≥12 weeks of omalizumab exposure. Three patients received treatments for AA, including intralesional corticosteroid injections, topical steroids and minoxidil, carpronium chloride solution, and liquid nitrogen therapy (15–17), and two of them continued omalizumab treatment. The patient with alopecia universalis showed no improvement in alopecia symptoms at 6-month follow-up after discontinuation of omalizumab, while others with AA continued omalizumab demonstrated partial recovery.

**Table 1:** Patients experienced hair loss or alopecia areata during omalizumab treatment.

References	Age (years)	Gender	Basic disease	Reason for use	Type of alopecia	Onset (weeks of using omalizumab)	Omalizumab discontinued/continued	Treatment	Duration	Outcome
Konstantinou et al. (13)	38	Female	/	CSU with angioedema	Telogen effluvium	4	Continued	/	3 to 4 months	Improved
	62	Female	/	CSU with angioedema	Telogen effluvium	4	Continued	/	3 to 4 months	Improved
	70	Female	Hashimoto’s thyroiditis	CSU with angioedema	Telogen Effluvium, alopecia areata	4	Continued	/	3 to 4 months	Improved
Noshela Ghazanfar and Thomsen (14)	27	Female	/	Three weeks history of urticaria	Hair loss without visible alopecia areata	12	Continued	/	2 months	Improved
Magen (15)	27	Male	/	CSU	Alopecia Areata	14	No data	Intralesional triamcinolone acetonide, 5 mg/mL every 4 weeks	No data	No data
Jin et al. (16)	34	Male	/	Asthma, nasal polyps	Alopecia areata	12	Continued	Topical minoxidil and steroids	Transient	Improved
Miyamoto et al. (17)	14	Female	Atopic dermatitis and seasonal allergic rhinitis	CSU, AD and seasonal allergic rhinitis	Alopecia areata	12 weeks of the second course	Continued	Topical steroids, carpronium chloride solution, and liquid nitrogen therapy	6 months	Improved
Numair et al. (18)	14	Male	/	Severe persistent asthma, allergic rhinitis, and allergic bronchopulmonary mycosis	Alopecia universalis	22	Discontinued	/	May be permanent	No significant improvement

CSU, chronic spontaneous urticaria; AD, atopic dermatitis.

## 4 FAERS database analysis

A total of 1,321 cases of alopecia associated with the use of omalizumab were identified from AE reports in the FAERS database, covering the period from 2004–2024. To minimize confounding effects from other suspected medications, 565 cases involving concomitant drugs were excluded, leaving 756 cases for focused analysis, (see Table 2). Among these 756 cases, 432 were reported by healthcare professionals and 319 by consumers. Age was not reported in 483 cases. Of the remaining 273 cases, ages ranged from 7 to 85 years (47.4 ± 16.87), with 14 cases under 18 years, 81 cases aged 18–40 years, 111 cases aged 41–60 years, and 67 cases over 60 years. Regarding gender distribution among the 756 cases, 119 were unspecified, 50 were men, and 587 were women.

**Table 2:** Clinical data of 756 omalizumab-related alopecia in the FAERS database.

Characteristics	Value (*n* = 756)
Gender, *n* (%)	
Male	50 (6.61)
Female	587 (77.65)
Not specified	119 (15.74)
Age (years), *n* (%)	
<18	14 (1.85)
18–40	81 (10.71)
41–60	111 (14.68)
>60	67 (8.86)
Not specified	483 (63.89)
Reporter type	
Healthcare professional	423 (57.14)
Consumer	319 (42.20)
Reason for use	
Urticaria/Angioedema	290 (38.36)
Asthma	191 (25.26)
Others	26 (3.44)
Unknown indication	249 (32.94)
Reactions	
Alopecia	742 (98.15)
Alopecia areata	7 (0.93)
Madarosis + alopecia	2 (0.26)
Diffuse alopecia	2 (0.26)
Alopecia universalis	1 (0.13)
Alopecia scarring	1 (0.13)
Alopecia; trichotillomania	1 (0.13)
Outcome in those only reported alopecia, *n* (%)	Value (*n* = 511)
Serious	15 (2.94)
Non-serious	496 (97.06)

The indications for omalizumab use in these patients included: 249 cases with unknown indication, 290 cases for urticaria/angioedema, 191 cases for asthma, four cases for asthma + urticaria, three cases each for nasal polyps and food allergy, two cases each for atopic dermatitis and mastocytosis, and one case each for mast cell activation syndrome, hypersensitivity + urticaria, atopic dermatitis + urticaria, eczema + hypersensitivity + urticaria, anaphylactic reaction + urticaria, asthma + fungal infection, asthma + atopic dermatitis, asthma + allergic bronchopulmonary aspergillosis, asthma + hypersensitivity, asthma + eczema, asthma + pneumonia, and allergy to arthropod sting. Among the 756 cases, the types of alopecia reported included seven cases of “Alopecia Areata,” two cases of “Madarosis; Alopecia,” two cases of “Diffuse Alopecia,” one case each of “Alopecia Universalis,” “Alopecia Scarring,” and “Alopecia; Trichotillomania,” while the remaining 742 cases were reported only as “Alopecia.” Additionally, 245 cases reported other AEs besides alopecia, with the most common being reactions already documented in the product labeling, including joint pain (38 cases), fatigue (34 cases), urticaria (31 cases), weight increased (21 cases), headache (20 cases), nausea (17 cases), paresthesia (15 cases), and injection site reactions (13 cases). Among the 511 cases reporting alopecia as the only AE, 15 were classified as “serious,” though no hospitalizations or death occurred.

## 5 Discussion

The drug labeling of omalizumab has undergone continuous revisions based on adverse risk information collected from post-marketing spontaneous reports and clinical use. Paying attention to adverse information associated with drug use is crucial, as it enhances patient safety in medication administration, improves the quality of healthcare, and promotes ongoing drug innovation and improvement (19). Medication-induced hair loss is a relatively rare type of adverse drug reaction. As novel targeted therapies are developed, the incidence of drug-induced alopecia is rising (20). Omalizumab is a biologic agent that targets IgE, and alopecia associated with it has been rarely reported. Enhancing awareness of this potential adverse effect can help optimize pre-treatment planning and patient counseling.

A variety of drugs such as cytotoxic drugs, biologics and immunomodulators can induce alopecia. Medication-induced alopecia primarily included anagen effluvium, telogen effluvium, AA, and scarring alopecia (20). Anagen effluvium is more common in patients using chemotherapeutic agents (21), and scarring alopecia is mostly linked to the use of epidermal growth factor receptor tyrosine kinase inhibitors (22). Telogen effluvium is the most common type of reversible drug-induced alopecia, possibly due to drug-induced abnormality in the normal hair cycle (23). AA is characterized by sudden, localized, non-scarring patchy hair loss that can occur in any hair-bearing region and may progress to totalis or universalis in severe cases. As an autoimmune disorder, the exact pathogenesis of AA remains unclear but involves genetically predisposed, environmentally triggered immune response targeting hair follicles. Potential mechanisms include loss of immune privilege in hair follicles, cytotoxic immune cell activation, and dysregulated follicular immune responses (24). Most cases of AA are idiopathic, but several triggers including medications, have been reported. Some scholars have used the FAERS database to analyze all cases reporting AA as an adverse event and identified monoclonal antibodies as the most frequently cited type of drugs associated with AA, such as TNF-α inhibitors and dupilumab, whereas omalizumab was not among the 30 most frequently used drugs (25). It has now been suggested that the downregulation of Th2 after dupilumab use may result in an abrupt skewing to Th1, promoting the pathogenesis of AA (26). However, in reality, dupilumab shows dual effects on AA, as it also be used as a treatment for AA due to reports of disease improvement with its use. This duality highlights our incomplete understanding of the pathogenesis of AA, which may exist of Th1-skewing and Th2-skewing AA subtypes (27), and suggests the complex mechanisms underlying monoclonal antibody-induced AA.

Here, we report a case of a 52-year-old woman with CSU who developed AA localized exclusively to the eyebrows after 12 weeks of omalizumab treatment. Although hair loss is listed among the AEs of omalizumab, there are no available data to estimate its frequency. Our literature review revealed extremely rare reports of alopecia during omalizumab treatment, with only eight patients, five of whom were women, with a mean age of 35.75 years. By analyzing the FAERS database, females constitute the vast majority of omalizumab associated alopecia cases, with the highest incidence observed in the 18–40 and 41–60 age groups. A cross-sectional analysis of medications used by patients reporting AA on the FAERS similarly demonstrated that approximately 75.6% of the reports were females, with the highest cases numbers in the 18–41 and 42–64 years age groups (25). A previous report on drug-induced AA indicated that about half of the affected patients were female and the mean age of onset was 39.5 years (28). A recent pharmacovigilance study of omalizumab using disproportionality analysis in the FAERS database also showed that the incidence of adverse drug events (ADEs) was significantly higher in women than in men, and this gender difference was particularly pronounced in ADEs with alopecia (29). These findings suggested that drug-induced AA/alopecia predominantly affected female patients and young to middle-aged populations. However, due to multiple potential confounding factors, such as underreporting and selection bias of the FAERS database, large-sample epidemiological studies are required to confirm these findings.

In our case, the patient continued omalizumab treatment after the onset of AA, with concurrent topical application of 0.03% tacrolimus ointment on the affected eyebrow areas. Her hair loss gradually improved over the following 16 weeks. Although literature suggested limited evidence supporting the efficacy of tacrolimus ointment for AA (30), it was selected due to the patient’s refusal of topical corticosteroids. Based on the current limited case reports, AA often occurred after 12 weeks of omalizumab use. Localized AA may represent a transient reaction that does not necessitate discontinuation of omalizumab, whereas alopecia universalis may portend a poorer prognosis. Notably, given the insufficient evidence for topical tacrolimus efficacy for AA, this case supported the possibility that such hair loss is self-limiting.

Potential mechanisms underlying omalizumab-induced alopecia may include the following: (1) Mast cells are important functional modulators of the follicular circulation (31). Omalizumab down-regulates the activity of mast cells, which may disrupt the normal hair growth cycle and lead to alopecia. Furthermore, mast cells can secrete molecules such as TNF-α and CCL5 that impact T-cell function (32), thereby potentially inducing AA. (2) Omalizumab may indirectly down-regulate the Th2 pathway by decreasing the level of free IgE, potentially amplifying the Th1 pathway, which may promote AA progression. (3) The increased total IgE levels observed after omalizumab treatment may play a role in the Th2-skewed immune characteristics of AA, as previous studies have reported increased total IgE levels in AA patients (33). In practice, however, it is difficult to determine a direct cause-effect relationship between omalizumab and new-onset alopecia. The fact that patients receiving omalizumab may have comorbid atopic background, which is thought to play an important pathogenic role in AA. Furthermore, some patients may be on multiple medications concurrently, makes it challenging to determine a definitive attribution. Our patient in this case denied personal and familial atopic background. However, we cannot completely rule out a potential association between the onset of AA and either CSU itself or the previous medications used. Employing standardized causality assessment tools (such as the Naranjo algorithm) may provide better insight into their relationship.

In conclusion, we report a rare case of eyebrow alopecia areata following omalizumab treatment and discuss the potential association between omalizumab and alopecia. Although direct attribution is difficult, we think that AA may represent a potential cutaneous AE with omalizumab use. Particular attention is warranted in female patients aged 18–60 years and patients receiving omalizumab therapy extending beyond 12 weeks. The clinical significance of this report lies in: (1) highlighting AA as a potential AE to omalizumab; (2) emphasizing the need for dynamic monitoring of hair alteration during biologic therapy; and (3) providing new insights into the role of IgE pathway in the pathogenesis of AA. This article aims to refine the safety profile of omalizumab by combining case report, literature review, and pharmacovigilance data. Even with its low incidence rate, awareness of this adverse event can inform clinical decision-making and patient management.

## Data Availability

The original contributions presented in the study are included in the article/supplementary material, further inquiries can be directed to the corresponding authors.
